# Identification of FOXP1 as a favorable prognostic biomarker and tumor suppressor in intrahepatic cholangiocarcinoma

**DOI:** 10.1186/s12885-024-11882-x

**Published:** 2024-01-26

**Authors:** Chenwei Tang, Hongkai Zhuang, Huanjun Tong, Xiaopeng Yu, Jialu Chen, Qingbin Wang, Xiaowu Ma, Bingkun Wang, Yonglin Hua, Changzhen Shang, Zhaohui Tang

**Affiliations:** 1 Department of Hepatobiliary Surgery, Sun Yat-Sen Memorial Hospital, Sun Yat-Sen University, Guangzhou, 510220 Guangdong Province China; 2 Department of General Surgery, Xinhua Hospital Affiliated to Medical College of Shanghai Jiaotong University, Shanghai, 200000 China

**Keywords:** Intrahepatic cholangiocarcinoma, Immunohistochemistry, Outcome prediction, Protein biomarker

## Abstract

**Background:**

Forkhead-box protein P1 (FOXP1) has been proposed to have both oncogenic and tumor-suppressive properties, depending on tumor heterogeneity. However, the role of FOXP1 in intrahepatic cholangiocarcinoma (ICC) has not been previously reported.

**Methods:**

Immunohistochemistry was performed to detect FOXP1 expression in ICC and normal liver tissues. The relationship between FOXP1 levels and the clinicopathological characteristics of patients with ICC was evaluated. Finally, in vitro and in vivo experiments were conducted to examine the regulatory role of FOXP1 in ICC cells.

**Results:**

FOXP1 was significantly downregulated in the ICC compared to their peritumoral tissues (*p* < 0.01). The positive rates of FOXP1 were significantly lower in patients with poor differentiation, lymph node metastasis, invasion into surrounding organs, and advanced stages (*p* < 0.05). Notably, patients with FOXP1 positivity had better outcomes (overall survival) than those with FOXP1 negativity (*p* < 0.05), as revealed by Kaplan–Meier survival analysis. Moreover, Cox multivariate analysis showed that negative FOXP1 expression, advanced TNM stages, invasion, and lymph node metastasis were independent prognostic risk factors in patients with ICC. Lastly, overexpression of FOXP1 inhibited the proliferation, migration, and invasion of ICC cells and promoted apoptosis, whereas knockdown of FOXP1 had the opposite role.

**Conclusion:**

Our findings suggest that FOXP1 may serve as a novel outcome predictor for ICC as well as a tumor suppressor that may contribute to cancer treatment.

**Supplementary Information:**

The online version contains supplementary material available at 10.1186/s12885-024-11882-x.

## Introduction

Intrahepatic cholangiocarcinoma (ICC, or iCCA) is a special type of primary liver cancer that originates from the intrahepatic bile duct [1]. ICC accounts for the second-highest incidence of all liver cancers after hepatocellular carcinoma (hCCA) [2]. The number of patients diagnosed with ICC has steadily risen over the past decades and is predicted to further increase in the coming years [3–5]. In America, a steady increase in ICC incidence has been reported in both men and women [6]. Radical surgery is generally regarded as the only way to achieve a complete cure and the first option for patients with ICC. Unfortunately, despite the significant advances in diagnosis and treatment, the outcomes of patients with ICC remain poor, with overall 5-year survival rates ranging from 23.6% to 33.9% [7–9]. Many clinical-pathological features, including tumor size, lymph node metastasis, and tumor differentiation status [10] influence the prognosis of ICC patients. Prognostic prediction is of great significance for clinical treatment strategies and could be performed by determining some specific molecules that are expressed in cancer [11]. Thus, a comprehensive understanding of the cellular microenvironment of ICC is critical for the identification of prognostic biomarkers and the development of more effective therapeutics.

The transcription factor forkhead box P1 (FOXP1) is a member of the Forkhead box (FOX) protein family, which shares a highly conserved forkhead DNA-binding domain. These proteins play vital roles in regulating transcription and are involved in cell differentiation, growth, embryogenesis, and longevity [12]. Studies have revealed a high degree of heterogeneity in the expression and function of FOXP1 in various malignancies, suggesting that it could be a key molecule and prognostic predictor [13, 14]. For instance, FOXP1 expression was significantly downregulated in the pancreatic ductal adenocarcinoma compared to that in the adjacent tissues, and a negative FOXP1 expression was associated with poor differentiation, lymph node metastasis, invasion of adjacent tissues, and a poor prognosis [15]. Similar results were found in both extrahepatic cholangiocarcinoma [16] and colorectal cancer [17]. FOXP1 was also reported to act as a tumor suppressor and negatively regulate immune responses, including cytokine and chemokine expression, in breast cancer [18]. FOXP1 knockdown was shown to promote tumor growth and invasion in lung adenocarcinoma [19], while FOXP1 overexpression can suppress cell migration and proliferation in rectal and prostate cancer cells [17, 20]. Moreover, FOXP1 was reported to be a valuable predictor of outcomes in different types of cancer [21–23]. However, what role FOXP1 played and whether it was associated with prognosis in the ICC remain unknown.

The present study aimed to analyze the relationship between FOXP1 expression and clinicopathological features, as well as prognosis, and initially explore the role of FOXP1 in ICC. The clinicopathological importance and prognostic value of FOXP1 in ICC were investigated. Immunohistochemistry was used to detect the expression of FOXP1 in the ICC and adjacent tissues. Furthermore, in vitro and in vivo experiments were conducted to determine the role of FOXP1 on the proliferation, invasion, migration, and apoptosis of ICC cells.

## Material and methods

### Patients, follow-up, and clinical samples

Patients who underwent radical hepatectomy at Xinhua Hospital (affiliated with the Shanghai Jiaotong University School of Medicine) and Sun Yat-sen Memorial Hospital from January 1, 2013 to December 12, 2019 were enrolled in this study. A total of 74 (54 for Xinhua Hospital and 20 for Sun Yat-sen Memorial Hospital) pathological sections of ICC and matched normal tissues (only 66 were obtained) were collected. Clinicopathological data, including tumor size, TNM stage, degree of differentiation, peripheral tissue invasion, vessel invasion, and lymph node metastasis, were manually collected. The pathological diagnoses of ICC, tumor staging, and degree of differentiation were determined as per the AJCC TNM staging system, 8th edition, and the World Health Organization criteria. The overall survival (OS, defined as the time from the day after surgery to the day of death) was obtained via phone call or written communication. The last follow-up was performed in November 2023, with a median follow-up time of 64.5 months (range, 28.5–96.0). The Ethics Committee of Xinhua Hospital (affiliated with the Shanghai Jiaotong University School of Medicine) and the Ethics Committee of Sun Yat-sen Memorial Hospital approved this study (no. XHEC-D-2021–047). Written informed consent was obtained from all of the enrolled patients.

### Immunohistochemistry

Rabbit anti-human FOXP1 monoclonal antibodies were purchased from Cell Signaling Technology (Danvers, MA, USA). Immunohistochemistry was performed using a commercially available kit (Sango Biotech, Shanghai, China), according to the manufacturer’s instructions. Briefly, paraffin-embedded tissues were cut into 5-μm-thick sections. Sections were deparaffinized in xylene and incubated with 3% H_2_O_2_ for 10 min. A microwave-thermal repair method was used to retrieve antigens. The sections were then incubated overnight at 4 °C with the primary anti-FOXP1 antibodies (1:100 dilution). After three washes with PBST, the sections were incubated with secondary antibodies for 30 min at room temperature. Finally, sections were revealed using DAB and counterstained with hematoxylin. Sections were finally dehydrated by immersion in different concentrations of ethanol and xylene, and were secured with neutral balsam. Two researchers evaluated 500 cells (first counting biliary epithelial cells) from 10 randomly selected fields per section, and determined, independently, the average percentage of positive cells. Tissues showing ≥ 25% positive cells were classified as positive, whereas the other cases were classified as negative. The intrahepatic bile duct epithelial cells and hepatocytes were considered adjacent normal tissues.

### Cell culture and transfection

Human HCCC-9810, RBE, QBC939, and HUCCT1 cells were obtained from the Cell Bank of the Chinese Academy of Sciences (Biological Industries; Shanghai, China) and cultured in RMPI 1640 medium or DMEM (Gibco BRL, Grand Island, NY, USA). The media were supplemented with 10% fetal bovine serum (FBS; Procell Life Science & Technology Co., Ltd., Wuhan, China) and cells were incubated under an atmosphere with 5% CO2 at 37 °C. The small interfering RNA (siRNA) targeting FOXP1 (siFOXP1) and control siRNA (siNC) were designed and chemically synthesized by the Gene Chemical Technology Co., Ltd. Plasmid vectors containing the FOXP1 overexpression sequence (Supplementary materials S 1, FOXP1 OE) or the negative control sequence (OE NC) were generated by Kidan Biosciences Co., Ltd. (Guangzhou, China) and used for cell transfection. Briefly, cells cultured in 6-well plates up to 80–90% of confluence were transfected with the different plasmids or siRNAs using Lipofectamine 3000 (Invitrogen, Carlsbad, CA, USA), according to the manufacturer’s instructions. Transfection efficiency was validated at 48 h after transfection. Cells were harvested after two days of incubation for use in the subsequent experiments, including cell function tests and RNA or protein extraction.

### Quantitative Real-Time Polymerase Chain Reaction (qRT-PCR)

Cells were harvested and total RNA was extracted using the EZ-press RNA Purification Kit (EZBioscience, Roseville, USA); the concentration was evaluated using a Nanodrop (ND-2000), and the 260/280 ratio was used to assess quality. The Evo M-MLV RT Premix was used to reverse-transcribe the RNA into cDNA for qRT-PCR analysis (Accurate Biotechnology Co., Ltd., Hunan, China). qRT-PCR was performed using SYBR® Green Pro Taq HS Premix (Accurate Biotechnology Co., Ltd., Hunan, China) to detect FOXP1 expression based on the 2^−ΔΔCT^ method. The amplification conditions were set up as follows: 45 cycles of denaturation for 10 s at 95 °C, annealing for 20 s at 55 °C, and extension for 20 s at 72 °C. The following primers were used: FOXP1 5’-TGGCATCTAATAAACCATCAGC-3’ (forward), and 5’-GGTCCACTCATCTTCGTCTCGA-3’ (reverse).

### Western blot

Cells were lysed with a commercially available reagent for western blotting and IP (Beyotime, Wuhan, China) on ice for 25 min, and centrifuged at 13,500 g for 30 min. The supernatant was collected and the protein concentration was measured using the bicinchoninic acid method (CWBIO, Beijing, China). SDS-PAGE gels (EpiZyme, Shanghai, China) were used for protein electrophoresis, followed by protein electrotransfer onto PVDF membranes (Roche Applied Science, Mannheim, Germany). The membranes were cut to probe with multiple antibodies against proteins of different molecular weights, and were incubated for 24 h at 4 °C with the primary antibodies at a dilution of 1:1000, followed by 2 h of incubation at room temperature with horse-radish peroxidase-conjugated secondary antibodies. Finally, using a G: BOX Chemi XT4 imager, protein bands were visualized via reaction with the Clarity Western ECL Substrate (Bio-Rad, Hercules, USA) The ImageJ software was used to measure the band densities. Tubulin was selected as the loading control. Supplementary materials S 2 lists all of the antibodies used in this study.

### Flow cytometry

The Annexin V-EV450/7-AAD apoptosis kit (Elabscience, Wuhan, China) was used to detect the extent of programmed cell death. Briefly, HCCC-9810 or HUCCT1 cells were centrifuged at 1000 rpm for 5 min and stained with Annexin V-V450 for 15 min in the dark and 7-AAD for 1 min, followed by flow cytometry analysis (BD FACSVerse, NYC, USA). The Flowjo (Version X10.0) software was used for the analysis.

### Cell proliferation assays

Cell viability was assessed using the colony formation assay and the cell counting Kit-8 (CCK-8). For the colony formation assay, transfected cells were seeded into 6-well plates (1500 cells per well for HCCC-9810; 2000 cells per well for HUCCT1), gently shaken, and cultured at 37 °C for 14 days. The culture medium was replaced every 3 days. Cells were then fixed with 4% paraformaldehyde for 15 min, and stained with 0.1% crystal violet for 10 min. The number of colonies (diameter > 30 μm) was counted and plotted as the mean ± standard deviation of three independent experiments. For the CCK-8, 2 × 10^3^ cells/well were seeded into 96-well plates. On days 1–5, the culture medium was replaced with the CCK-8 working solution (DMEM:CCK-8 = 100:1), and the plates were incubated in the dark for 2 h at 37 °C with 5% CO2. The absorbance (OD450) was finally measured using a microplate reader (Thermo MK3, Thermo Fisher Scientific, Waltham, USA).

### Cell migration assays

Transwell and wound healing experiments were performed to assess cell migration. For the former, in each upper chamber of the Transwell plates (Bio-Filtration Co., Ltd., Guangzhou, China), 4 × 10^4^ cells mixed with 200 μL serum-free DMEM were seeded; 500 μL DMEM supplemented with 100 μL FBS was added to each lower chamber. After 48 h of culture, the upper chambers were collected, fixed with 4% paraformaldehyde for 15 min, and stained with 0.3% crystal violet for 10 min. The migrated cells were photographed and counted under a microscope in three randomly selected fields. For the wound-healing assay, 2 × 10^5^ cells/well were seeded into 6-well plates. After culturing to 100% confluence, a wound was made in the cell monolayer with a sterile 1-mL plastic pipette tip. The cells were then cultured for 24 h in culture medium supplemented with 2% FBS. Images of the wounds were captured at 0 h and 24 h using a photomicroscope (Olympus IX73, Tokyo, Japan) to assess cell migration. The Image J software (National Institutes of Health, Bethesda, MD, USA) was used to compute the wound areas.

### Cell invasion assay

For cell invasion analysis, Transwell plates (Bio-Filtration Co., Ltd., Guangzhou, China) were used. Except for the addition of Matrigel (Corning, NY, USA) to the upper chamber a day before cell seeding, the remaining steps of this protocol were the same as those described above.

### Animal study

HCCC-9810 cells were stably transfected by lentivirus with FOXP1 overexpression or knockdown (Gene Chemical Technology Co., Ltd.) and were used to construct subcutaneous xenograft models. A total of 12 five-week-old BALB/c nude mice were randomized into three groups. The mice were subcutaneously injected with 150 μL of a PBS-Matrigel mixture (PBS: Matrigel = 2:1) containing 5 × 10^6^ cells. The tumor volume (length × width^2^ × 0.5) was measured and recorded every 6 days. The mice were sacrificed by dislocation after being anesthetized by intraperitoneal injection of 1% sodium pentobarbital (50 mg/kg), and the tumors were isolated, weighed, and fixed in 4% paraformaldehyde. The Animal Ethics Committee of Sun Yat-sen University approved the animal experiments, and the study was conducted in the Animal Center of the university.

### Statistical analysis

All data were analyzed using the SPSS 21.0 (Version 21.0, SPSS Inc., Chicago, IL, USA) and the GraphPad Prism (version 8.0; San Diego, CA, USA) software. The relationship between FOXP1 expression in ICC tissues and clinicopathological features was evaluated using either the χ2 or the Fisher’s exact test. The nonparametric Mann–Whitney U test was applied to compare the overall survival between different groups. Survival curves were drawn using the Kaplan–Meier univariate survival analysis and the log-rank test. The Cox proportional hazards model was used for multivariate analysis with a 95% confidence interval. Statistical significance was set at *p* < 0.05.

## Results

### FOXP1 is downregulated in ICC

**Fig. 1:**
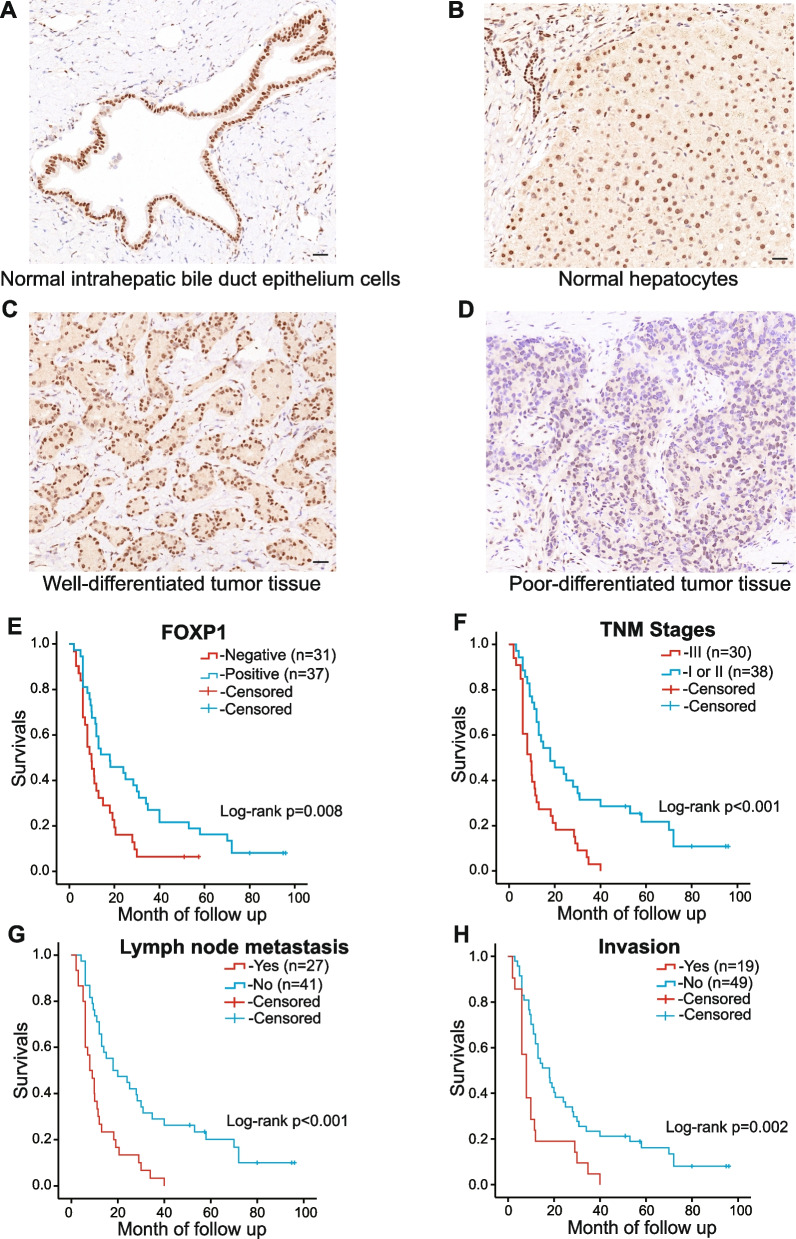
Immunohistochemistry staining of FOXP1 in ICC and adjacent normal tissues and the Kaplan–Meier analysis. **A, B** Positive expression of FOXP1 in the adjacent noncancerous tissues (intrahepatic bile duct epithelium and hepatocytes, respectively).** C** Positive expression of FOXP1 in well-differentiated ICC. **D** Negative expression of FOXP1 in poorly differentiated ICC. Pictures were taken at a magnification of 200 × , scale bar = 50 μm. **E–H** Kaplan–Meier plots of the overall survival of patients grouped by FOXP1 expression, TNM stages, lymph node metastasis, and organ invasion

As shown by immunohistochemistry staining, FOXP1 was mainly located in the nucleus and occasionally in the cytoplasm of cells. The positive FOXP1 expression was mostly observed in the intrahepatic bile duct epithelium and hepatocytes, and occasionally in the well-differentiated tumor tissues (Fig. 1A-D). The positive expression rate of FOXP1 in the ICC tissues was 54.1%, significantly lower than that in the peritumoral tissues (75.8%; *p* = 0.007; Table 1). Notably, positive expression of FOXP1 was observed in the normal intrahepatic bile duct epithelial cells and hepatocytes of the paracancerous tissues. Consistent with these results, microscopic examination confirmed that almost all the epithelial cells of intrahepatic bile ducts expressed FOXP1. Meanwhile, ICC tissues with negative expression of FOXP1 were often characterized by poor differentiation, irregular cell arrangement, loss of polarity, and rare adenoid structures (Fig. 1D).

**Table 1:** Expression of FOXP1 in ICC tissues and peritumoral tissues

Tissue types	FOXP1 expression		Total	*p*
	Negative (%)	Positive (%)		
ICC	34(45.9%)	40(54.1%)	74(100%)	*p* = 0.007
peri-ICC^a^	16(24.2%)	50(75.8%)	66(100%)	

*Note*: ^a^Peritumoral tissues

### Lower FOXP1 expression is related to more aggressive ICC features

We further evaluated the association between FOXP1 expression and the clinicopathological data (Table 2). The positive rate of FOXP1 in moderately or highly differentiated tissues was significantly higher than that in poorly differentiated tissues (*p* < 0.05). FOXP1 expression was also negatively correlated with lymph node metastasis (*p* < 0.01). Similarly, cancers that had invaded nearby organs had significantly lower levels of FOXP1 expression than those that had not (*p* < 0.05). The expression of FOXP1 was also correlated with the TNM stage (*p* < 0.01). Tumors staged as TNM III showed lower levels of FOXP1 than those staged as TNM I or II. However, there was no significant correlation between FOXP1 levels and other pathological features, such as sex, age, bile duct stones, hepatitis, tumor diameter, star lesions, or micro-nerve infiltration (*p* > 0.05).

**Table 2:** Correlations of FOXP1 expression with the clinicopathological characteristics of ICC

Clinicopathological characteristics	Case number(n)	FOXP1		
		Positive number (%)	Χ^2^	*p*
Age (years)				
≥ 63	31	17(54.8)	0.013	0.908
< 63	43	23(53.5)		
Sex				
Male	34	21(61.8)	1.506	0.220
Female	40	19(47.5)		
Differentiation				
Moderately-well	48	31(64.6)	6.098	**0.014**
Poorly	26	9(34.6)		
Pathogeny				
Hepatitis	12	7(58.3)	0.853	0.356
Stones	5	3(60.0)		
History of biliary surgery	4	3(75.0)		
Others	56	28(50.0)		
Tumor size (cm)				
< 5	21	9(42.9)	1.480	0.224
≥ 5	53	31(58.5)		
Lymph node metastasis				
Yes	29	10(34.5)	7.355	**0.007**
No	45	30(66.7)		
Invasion				
YES	21	7(33.3)	5.069	**0.024**
No	53	33(66.3)		
TNM Stages				
I-II	42	29(69.0)	8.792	**0.003**
III	32	11(34.4)		
Star lesion				
Yes	28	14(50.0)	0.298	0.585
No	46	26(56.5)		
Micronerve infiltration				
Yes	14	8(57.1)	0.066	0.797
No	60	32(53.3)		
Vessel invasion				
Yes	12	8(66.7)	0.917	0.338
No	62	32(51.6)		

### Low FOXP1 expression is correlated with an unfavorable prognosis in ICC

The prognostic information of patients with ICC was statistically analyzed. Six patients were lost to follow-up. Of the patients who were followed up, 40 died within a year, 20 lived for more than 1 year, and 8 lived for more than 3 years. Kaplan–Meier survival analysis revealed that patients with lymph node metastasis (*p* < 0.01), invasion events (*p* < 0.05), and advanced tumor stage (*p* < 0.01) had worse OS outcomes (Table 3, Fig. 1E-H). Additionally, the OS median of FOXP1-positive patients was higher than that of FOXP1-negative patients (18 months vs. 9 months, *p* < 0.05). Moreover, multivariate Cox regression analysis revealed that invasion of nearby organs, lymph node metastasis, and advanced TNM stage negatively correlated with the overall survival rate and positively correlated with the mortality rate (Table 4). High FOXP1 expression was positively correlated with the overall survival rate and negatively correlated with mortality in patients with ICC (Table 4). Overall, these results suggest that FOXP1 is an independent prognostic factor for patients with ICC.

**Table 3:** Correlations of clinicopathological characteristics and FOXP1 expression with the overall survival in patients with ICC

Group	Case number(n)	Overall survival (moth)	Mann–Whitney U	*p*
Age (years)				
≥ 63	28	12(2–95)	532	0.727
< 63	40	13(2–96)		
Sex				
Male	33	14(2–95)	534	0.589
Female	35	18(2–96)		
Differentiation				
Moderately-well	45	13(2–96)	514	0.522
Poorly	23	12(2–95)		
Hepatitis				
Yes	10	11(2–30)	238	0.362
No	58	15(2–96)		
Tumor size (cm)				
< 5	19	15(3–96)	444	0.768
≥ 5	49	14(2–95)		
Lymph node metastasis				
Yes	27	10(2–77)	359	**0.015**
No	41	18(2–96)		
Invasion				
YES	19	11(2–37)	260	**0.005**
No	49	20(2–96)		
TNM Stages				
I-II	38	21(2–96)	312	**0.001**
III	30	11.(2–40)		
Star lesion				
Yes	26	12(2–58)	437	0.168
No	42	16(2–96)		
Micronerve infiltration				
Yes	12	13(2–80)	375	0.729
No	56	14(2–96)		
Vessel invasion				
Yes	10	11(2–34)	253	0.521
No	58	14(2–96)		
FOXP1				
+	37	18(2–96)	373	**0.013**
-	31	9(2–77)

**Table 4:** Multivariate cox regression analysis of overall survival in patients with ICC

Group	Factors	β	SE	Wald	*p*	RR	95.0% CI	
							Lower	Upper
TNM Stages	I、II/III	0.998	0.328	9.240	0.002	2.714	1.426	5.165
invasion	Yes/No	1.109	0.335	9.261	0.002	2.771	1.437	5.342
lymph node metastasis	Yes/No	0.760	0.382	3.948	0.047	2.138	1.010	4.524
FOXP1	±	-0.611	0.296	4.272	0.039	0.543	0.304	0.969

*Abbreviations*: *β* regression coefficients, *SE* Standard error, *RR* Relative risk, *CI* confidence interval, *TNM* tumor–node–metastasis

### FOXP1 works as a tumor suppressor of ICC both in vitro and in vivo

**Fig. 2:**
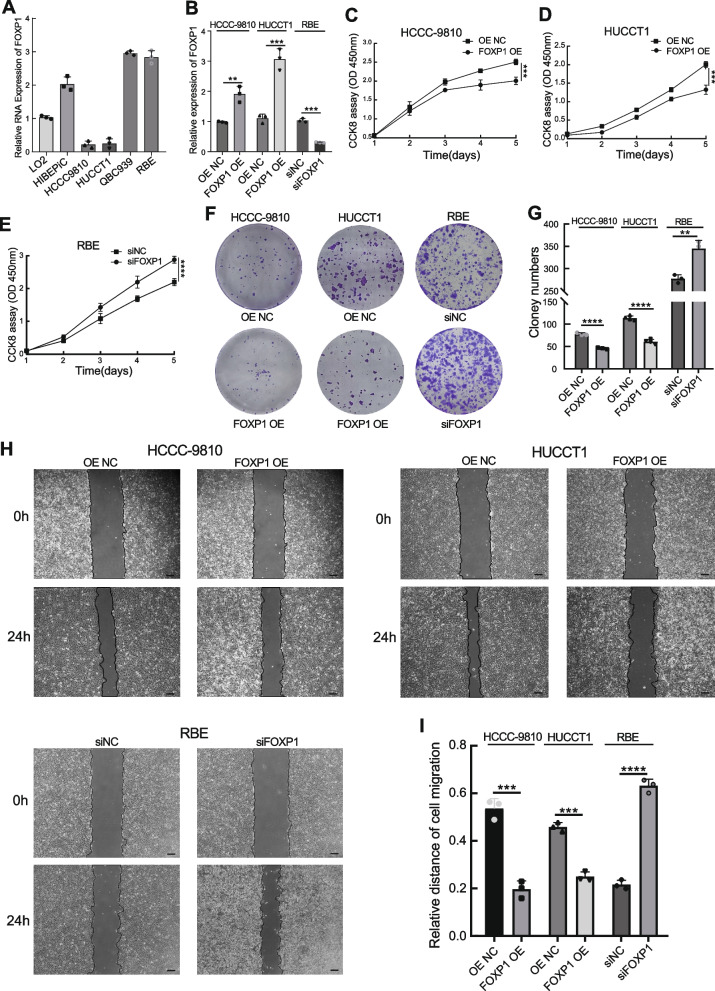
Effect of FOXP1 on the proliferation and migration of ICC cells. **A** FOXP1 expression in different ICC cell lines. **B** Validation of the FOXP1 overexpression or knockdown in indicated cells. **C-E** Growth curves of the indicated groups based on CCK-8 assays. **F, G** Colony formation assays of the FOXP1 upregulated or downregulated cells. Representative images of the colonies and quantitative statistical chart are shown. **H, I** Wound-healing assays were used to assess the cell migratory ability of indicated cells. scale bar = 200 μm. Data are presented as means ± SD of three independent experiments. **p < 0.01, ***p < 0.001, ****p < 0.0001

**Fig. 3:**
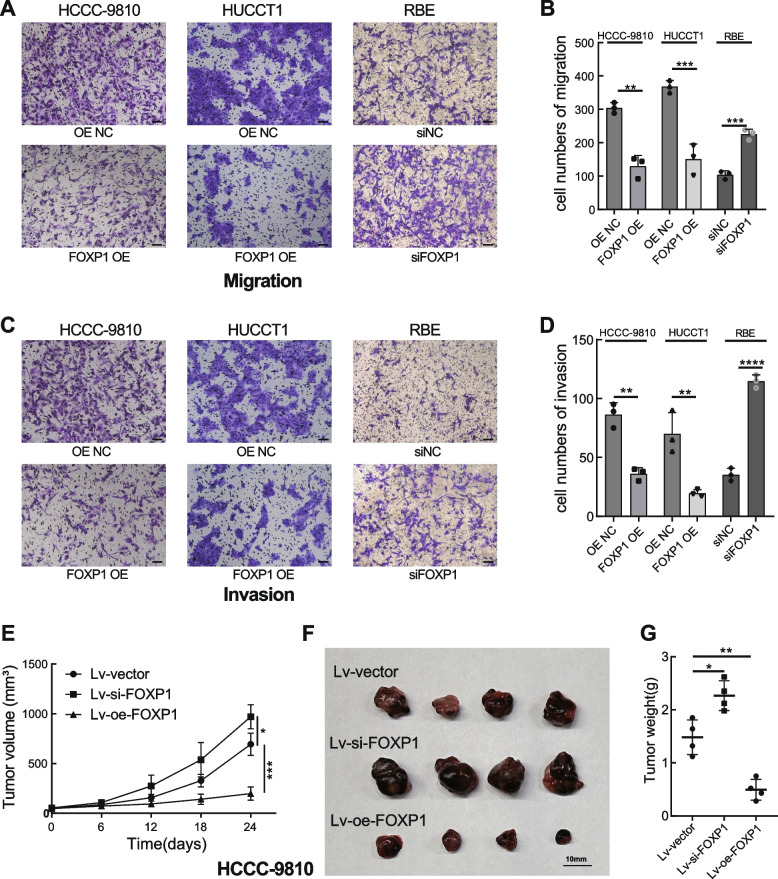
Impact of FOXP1 on ICC cell migration and invasion in vitro and growth in vivo. **A-D** Representative images of the successfully migrated or invaded cells and the quantitative statistical chart of the indicated cells. Scale bar = 100 μm. **E** Growth curves of subcutaneous tumors in nude mice. (n = 4). **F, G** Outlook and weight of the subcutaneous xenografts dissected from the mice at the endpoint. Data are presented as means ± SD of three independent experiments. *p < 0.05, **p < 0.01, ***p < 0.001

**Fig. 4:**
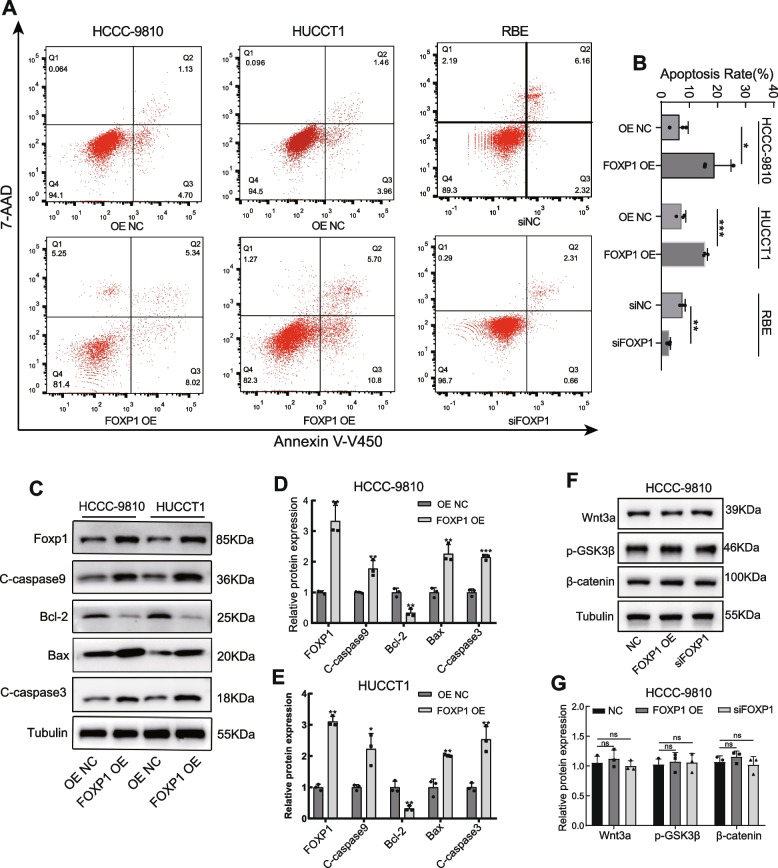
Apoptosis detection and validation of the Wnt/β-catenin signal pathway. **A**, **B** Apoptosis levels of the different groups of ICC cells, as per Annexin V-EV450/7-AAD flow cytometry analysis. **C-E** Western blot analysis of different apoptosis-related proteins in FOXP1-overexpressed (HCCC-9810 and HUCCT1) cells and the control cells. **F**-**G** Western bolt of key proteins in the Wnt/β-catenin pathway. Tubulin was used as the loading control. Data are presented as means ± SD of three independent experiments. *p < 0.05. **p < 0.01, ***p < 0.001

In order to explore the regulatory role of FOXP1, several ICC cell lines were subjected to expression determination of FOXP1. Based on qRT-PCR validation (Fig. 2A), HCCC-9810 and HUCCT1 cells (lower FOXP1 expression) were transfected with a FOXP1 overexpression plasmid, while RBE cells (high FOXP1 expression) were used for siRNA treatment targeting FOXP1. Both significant upregulation in the FOXP1 OE group and knockdown in the siFOXP1 group were observed (Fig. 2B). As a result, upregulation of FOXP1 significantly suppressed HCCC-9810 and HUCCT1 cell proliferation, while FOXP1 downregulation promoted RBE cell growth, as verified by CCK-8 and colony formation assays (Fig. 2C–G). As per the Transwell and wound healing experiments, cellular migration and invasion were significantly impaired or increased after FOXP1 overexpression or knockdown, respectively (Fig. 2H, I and Fig. 3A-D). Moreover, the animal study demonstrated that depletion of FOXP1 significantly increased the tumor growth rate, and FOXP1 upregulation exhibited the opposite trend when compared with the control group (Fig. 3E-G). Importantly, flow cytometry analysis showed that the proportion of apoptotic cells was significantly higher in the FOXP1 OE group than that in the control group, whereas a reduction of apoptosis was detected in cells with siFOXP1 (Fig. 4A, B). Consistent with this result, western blotting also revealed that the apoptosis-related proteins BAX, cleaved-Caspase3, and cleaved-Caspase9 were significantly upregulated, whereas that of anti-apoptotic BCL-2 was significantly downregulated in ICC cells overexpressing FOXP1 (Fig. 4C-E). We further explored the possible mechanism by which FOXP1 inhibits ICC cell progression. Since the Wnt/β-catenin signaling pathway was previously reported as a vital regulatory factor in cholangiocarcinoma [24–26], the key pathway-related proteins, including Wnt3a, phosphorylated GSK3β, and β-catenin, were detected in HCCC-9810 cells after FOXP1 overexpression or knockdown. Unfortunately, the Wnt/β-catenin signaling pathway was not significantly altered by FOXP1 interference (Fig. 4F, G). Altogether, these findings demonstrate that FOXP1 overexpression inhibits ICC progression via the promotion of apoptosis, suggesting a tumor suppressor role for FOXP1.

## Discussion

ICC is a special primary liver cancer characterized by high malignancy, quick tumor progression, and an extremely poor prognosis [27]. Currently, effective targets for guiding precision medicine and predicting prognosis accurately are, however, still hardly available. A thorough investigation into the intracellular abnormalities of specific molecules may contribute to the identification of new therapeutic targets and outcome prediction. FOXP1 is an essential tumor-regulatory molecular that has been widely studied in various cancer types [14, 28]. For the first time, the present study explored the unknown role of FOXP1 in ICC and yielded a preliminary glimpse into FOXP1-induced ICC suppression. As verified in a relatively large cohort (a total of 74 patients), FOXP1 was found to be downregulated in ICC tissues, and negative FOXP1 expression is an independent prognostic risk factor for OS. The subsequent function experiments revealed the inhibitory role of FOXP1 in ICC, both in vitro and in vivo. Taken together, FOXP1 may serve as a prognosis predictor and a novel possible therapeutic target for patients with ICC.

FOXP1, a member of the forkhead box protein family, is widely expressed in human tissues and plays a role in embryonic development, immune regulation, and cancer progression [28, 29]. Functionally, FOXP1 acts as a tumor suppressor in some solid tumors but also plays oncogenic roles, particularly in hematological malignancies [30]. For instance, downregulation of FOXP1 is associated with aggressive tumor behavior, resulting in poor prognosis in colorectal neoplasia [17], prostate cancer [20], endometrial [31], ovarian cancer [32], and renal cell carcinoma [33]. As for hematological malignancies, however, FOXP1 plays an oncogenic role and is associated with malignant progression and a poor prognosis [34]. Despite the many studies on the role of FOXP1 in other cancers, its role in ICC was first revealed by the present study. We found that FOXP1 upregulation inhibits the growth, migration, and invasion of ICC cells in vitro and promotes their apoptosis. However, the underlying mechanism by which FOXP1 works in ICC has not been elucidated previously.

The mechanisms of FOXP1-mediated regulation of tumor growth are multilayered and multidirectional, including those associated with tumor immunity [35], some lncRNAs [36], and cancer-related signal pathways [37]. For example, it has been observed that FOXP1 inhibits the expression of the interleukin-7 (IL-7) receptor α chain (IL-7Rα) in CD8 + T cells and phosphorylates MEK and ERK to exert an anti-tumor effect [38]. FOXP1 was also reported to act as a tumor suppressor via the negative regulation of the expression of IL-7 in tumor-specific CD4 + T helper cells 7 (Th9) [39]. In order to understand how FOXP1 inhibits ICC progression, we initially evaluated its potential role in the regulation of apoptosis. The apoptosis-related proteins (BAX, cleaved-Caspase3, and cleaved-Caspase9) were elevated, and the anti-apoptotic protein BCL-2 was downregulated after overexpression of FOXP1, suggesting that cell apoptosis may be a part of the FOXP1-induced ICC suppression mechanism. Since the Wnt/β-catenin pathway was thought to involve cholangiocarcinoma progression and apoptosis [25], this possibility was subsequently explored. It turns out that FOXP1 did not change the activity of the Wnt/β-catenin signal pathway. To sum up, except for the apoptosis-promoting function, the mechanism by which FOXP1 inhibits ICC progression is still unclear. This is one of the biggest limitations of our study, and we intend to continue investigating it in our future research.

Unlike extrahepatic cholangiocarcinoma or gallbladder cancer, ICC localizes in the liver and is contiguous to hepatocytes. ICC is considered to originate from the epithelial cells of the intrahepatic bile ducts. However, increasing evidence suggests that ICC may have numerous origins, including the surrounding gland cells, hepatocytes, and hepatic pluripotent stem cells [40, 41]. Because of the possibility of multiple sources, this study defined peritumoral tissues as adjacent bile duct epithelial cells and hepatocytes. Notably, in our quantitative microscopical analyses, we initially counted all normal bile duct epithelial cells. Additionally, we discovered that the expression of FOXP1 in bile duct cells was mostly the same as that in hepatocytes (Fig. 1B), implying that ICC may have multiple cell origins.

In summary, as determined by IHC staining of tissue sections from patients with ICC, FOXP1 was firstly identified to be downregulated in ICC. Patients with negative FOXP1 expression are usually characterized by aggressive features such as advanced stages, poor differentiation, and lymph node metastasis. Survival analysis revealed that patients with FOXP1 positive outlived those with FOXP1 negative, and negative FOXP1 expression was an independent prognostic risk factor for OS. Additionally, FOXP1 overexpression inhibited the proliferation, migration, and invasion of ICC cells and promoted their apoptosis, as verified by function experiments. These results provide preliminary evidence that FOXP1 is an ICC inhibitor and can be utilized to approximately predict prognosis. Nevertheless, there are certain shortcomings that need to be addressed in follow-up research, like a small number of enrolled patients and an unclear exploration of mechanisms.

## Conclusion

Overall, FOXP1 was identified as a newly developed tumor suppressor that exhibits high value in predicting the biological behavior and prognosis of ICC and is a novel possible therapeutic target.

### Supplementary Information

**Additional file 1**: **S1.** FOXP1 overexpression sequence. **S2.** Antibody used in this study. **S3.** The original images of the Western Blot.

## Data Availability

All data used to support the findings of this study are available from the corresponding author upon request.
