# Supplementary material for: Identification of FOXP1 as a favorable prognostic biomarker and tumor suppressor in intrahepatic cholangiocarcinoma
Source: BMC Cancer. 2024 Jan 26;24:137. doi: 10.1186/s12885-024-11882-x (PMC10811915; doi:10.1186/s12885-024-11882-x)
Supplement: Supplementary file 1 — Additional file 1: S1. FOXP1 overexpression sequence. S2. Antibody used in this study. S3. The original images of the Western Blot. [file 12885_2024_11882_MOESM1_ESM.docx]

**Supplementary materials**

**S1. FOXP1 overexpression sequence**

ATGATGCAAGAATCTGGGACTGAGACAAAAAGTAACGGTTCAGCCATCCAGAATGGGTCGGGCGGCAGCAACCACTTACTAGAGTGCGGCGGTCTTCGGGAGGGGCGGTCCAACGGAGAGACGCCGGCCGTGGACATCGGGGCAGCTGACCTCGCCCACGCCCAGCAGCAGCAGCAACAGGCACTTCAGGTGGCAAGACAGCTCCTTCTTCAGCAGCAACAGCAGCAGCAAGTTAGTGGATTAAAATCTCCCAAGAGGAATGACAAACAACCAGCTCTTCAGGTTCCCGTGTCAGTGGCTATGATGACACCTCAAGTTATCACTCCCCAGCAAATGCAGCAGATCCTCCAGCAACAAGTGCTGAGCCCTCAGCAGCTCCAGGTTCTCCTCCAGCAGCAGCAGGCCCTCATGCTTCAACAGCAGCAGCTTCAAGAGTTTTATAAAAAACAACAGGAACAGTTGCAGCTTCAACTTTTACAACAACAACATGCTGGAAAACAGCCTAAAGAGCAACAGCAGGTGGCTACCCAGCAGTTGGCTTTTCAGCAGCAGCTTTTACAGATGCAGCAGTTACAGCAGCAGCACCTCCTGTCTTTGCAGCGCCAAGGCCTTCTGACAATTCAGCCCGGGCAGCCTGCCCTTCCCCTTCAACCTCTTGCTCAAGGCATGATTCCAACAGAACTGCAGCAGCTCTGGAAAGAAGTGACAAGTGCTCATACTGCAGAAGAAACCACAGGCAACAATCACAGCAGTTTGGATCTGACCACGACATGTGTCTCCTCCTCTGCACCTTCCAAGACCTCCTTAATAATGAACCCACATGCCTCTACCAATGGACAGCTCTCAGTCCACACTCCCAAAAGGGAAAGTTTGTCCCATGAGGAGCACCCCCATAGCCATCCTCTCTATGGACATGGTGTATGCAAGTGGCCAGGCTGTGAAGCAGTGTGCGAAGATTTCCAATCATTTCTAAAACATCTCAACAGTGAGCATGCGCTGGACGATAGAAGTACAGCCCAATGTAGAGTACAAATGCAGGTTGTACAGCAGTTAGAGCTACAGCTTGCAAAAGACAAAGAACGCCTGCAAGCCATGATGACCCACCTGCATGTGAAGTCTACAGAACCCAAAGCCGCCCCTCAGCCCTTGAATCTGGTATCAAGTGTCACTCTCTCCAAGTCCGCATCGGAGGCTTCTCCACAGAGCTTACCTCATACTCCAACGACCCCAACCGCCCCCCTGACTCCCGTCACCCAAGGCCCCTCTGTCATCACAACCACCAGCATGCACACGGTGGGACCCATCCGCAGGCGGTACTCAGACAAATACAACGTGCCCATTTCGTCAGCAGATATTGCGCAGAACCAAGAATTTTATAAGAACGCAGAAGTTAGACCACCATTTACATATGCATCTTTAATTAGGCAGGCCATTCTCGAATCTCCAGAAAAGCAGCTAACACTAAATGAGATCTATAACTGGTTCACACGAATGTTTGCTTACTTCCGACGCAACGCGGCCACGTGGAAGAATGCAGTGCGTCATAATCTTAGTCTTCACAAGTGTTTTGTGCGAGTAGAAAACGTTAAAGGGGCAGTATGGACAGTGGATGAAGTAGAATTCCAAAAACGAAGGCCACAAAAGATCAGTGGTAACCCTTCCCTTATTAAAAACATGCAGAGCAGCCACGCCTACTGCACACCTCTCAATGCAGCTTTACAGGCTTCAATGGCTGAGAATAGTATACCTCTATACACTACCGCTTCCATGGGAAATCCCACTCTGGGCAACTTAGCCAGCGCAATACGGGAAGAGCTGAACGGGGCAATGGAGCATACCAACAGCAACGAGAGTGACAGCAGTCCAGGCAGATCTCCTATGCAAGCCGTGCATCCTGTACACGTCAAAGAAGAGCCCCTCGATCCAGAGGAAGCTGAAGGGCCCCTGTCCTTAGTGACAACAGCCAACCACAGTCCAGATTTTGACCATGACAGAGATTACGAAGATGAACCAGTAAACGAGGACATGGAGTGA

**Targeted sequence of FOXP1 siRNA：**CAGCAAGTTAGTGGATTAAAATC

**S2. Antibody used in this study**

| **Antibody/Working dilution** | **Source** |
| --- | --- |
| FOXP1 (1:1000) | 4402T, Cell Signaling Technology |
| Caspase-9 (1:1000) | 9502T, Cell Signaling Technology |
| BAX (1:1000) | 5023T,Cell Signaling Technology |
| BCL-2 (1:1000) | 60178-1-Ig, Proteintech |
| Caspase-3 (1:1000) | A19654,ABclonal Technology |
| Tubulin (1:1000) | 10068-1-AP, Proteintech |
| β-Catenin (1:1000) | A0316, ABclonal Technology |
| WNT3A (1:1000) | A0642, ABclonal Technology |
| p-GSK3β (phospho S9) | ab107166, abcam |

**S3. The original images of the Western Blot.
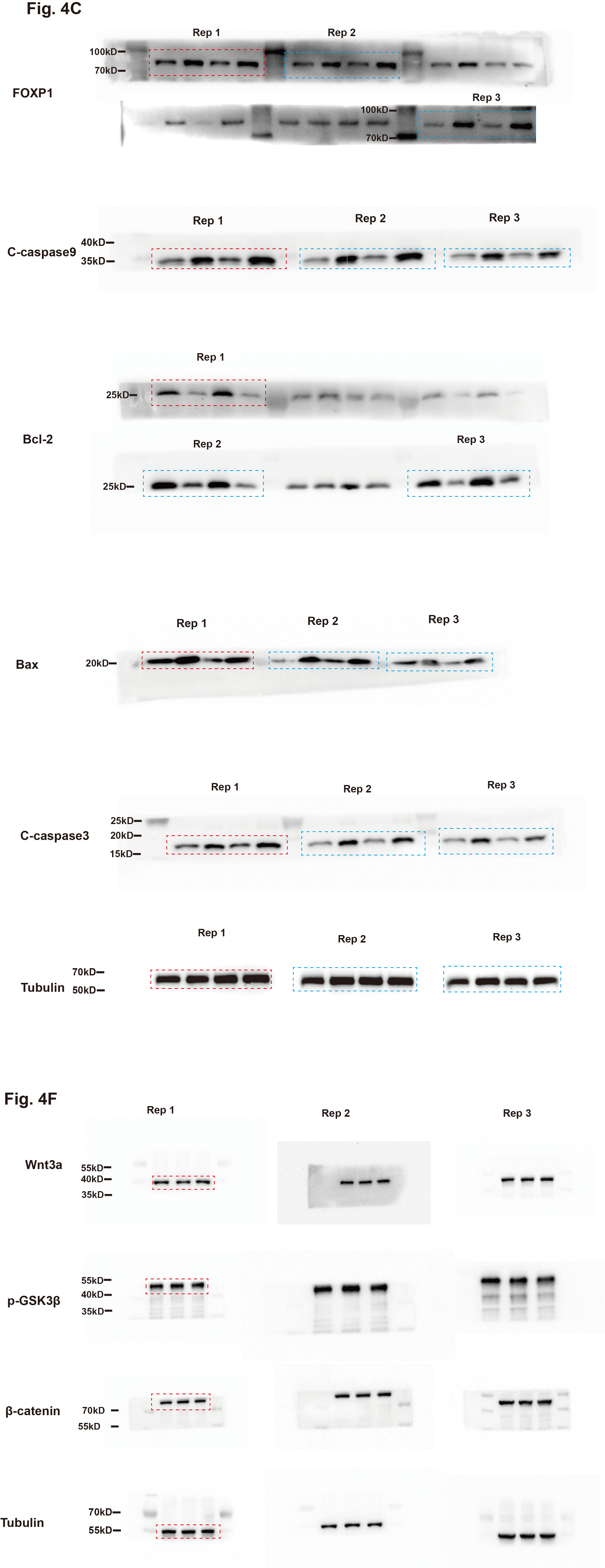
**

**
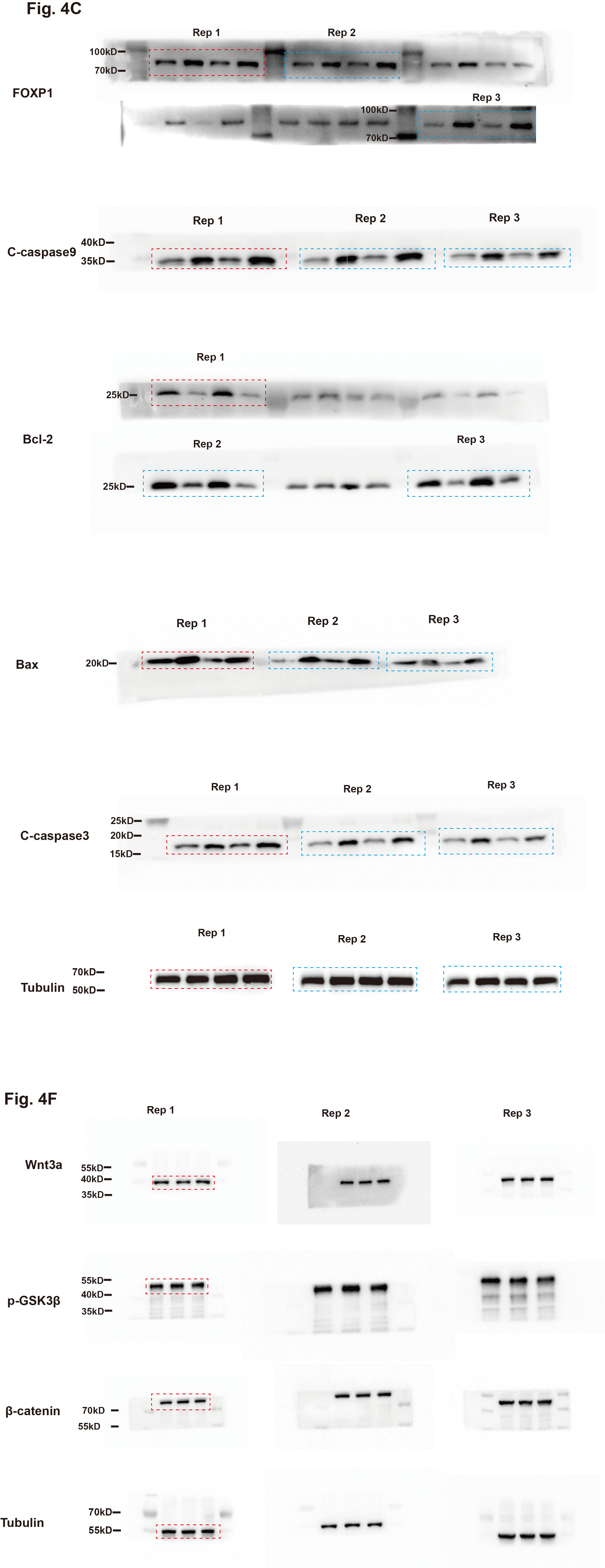
**
